# Expanding Avenue of Fast Neutron Mediated Mutagenesis for Crop Improvement

**DOI:** 10.3390/plants8060164

**Published:** 2019-06-10

**Authors:** Surbhi Kumawat, Nitika Rana, Ruchi Bansal, Gautam Vishwakarma, Sayaji T. Mehetre, Bikram Kishore Das, Manish Kumar, Satish Kumar Yadav, Humira Sonah, Tilak Raj Sharma, Rupesh Deshmukh

**Affiliations:** 1 National Agri-food Biotechnology Institute (NABI), Mohali, Punjab 140308, India; surbhikumawat002@gmail.com (S.K.); siesta.nitika@gmail.com (N.R.); ruchibansal18@gmail.com (R.B.); biohuma@gmail.com (H.S.); 2 Nuclear Agriculture & Biotechnology Division, Bhabha Atomic Research Centre, Mumbai 400085, India; gtmvish@barc.gov.in (G.V.); smehetre@barc.gov.in (S.T.M.); bkdas@barc.gov.in (B.K.D.); 3 Department of Seed Science and Technology, College of Horticulture, Dr. Yashwant Singh Parmar University of Horticulture and Forestry, Nauni, Solan, Himachal Pradesh 173230, India; drmanishuhf@rediffmail.com; 4 National Bureau of Plant Genetic Resources, New Delhi 110012, India; satish.yadav1@icar.gov.in

**Keywords:** induced mutagenesis, fast neutron irradiation, mutation mapping, deletagene, next-generation sequencing technologies, reverse genetics

## Abstract

Fast neutron (FN) radiation mediated mutagenesis is a unique approach among the several induced mutagenesis methods being used in plant science in terms of impacted mutations. The FN mutagenesis usually creates deletions from few bases to several million bases (Mb). A library of random deletion generated using FN mutagenesis lines can provide indispensable resources for the reverse genetic approaches. In this review, information from several efforts made using FN mutagenesis has been compiled to understand the type of induced mutations, frequency, and genetic stability. Concerns regarding the utilization of FN mutagenesis technique for a plant with different level of ploidy and genome complexity are discussed. We have highlighted the utility of next-generation sequencing techniques that can be efficiently utilized for the characterization of mutant lines as well as for the mapping of causal mutations. Pros and cons of mapping by mutation (MutMap), mutant chromosome sequencing (MutChromSeq), exon capture, whole genome sequencing, MutRen-Seq, and different tilling approaches that can be used for the detection of FN-induced mutation has also been discussed. Genomic resources developed using the FN mutagenesis have been catalogued wooing to meaningful utilization of the available resources. The information provided here will be helpful for the efficient exploration for the crop improvement programs and for better understanding of genetic regulations.

## 1. Introduction

Induced mutagenesis is an indispensable tool for the creation of new alleles which can be explored for crop improvement. Mutagenesis is a potent method for bordering genetic variability in different species. It has great importance, particularly where natural sources for the genetic variations are limited. Domestication process that has enhanced the yield potential of crop plant by many folds has also greatly affected the genetic diversity. Such limited genetic diversity in the cultivated species usually create a bottleneck for crop improvement. In this regard, mutagenesis approaches look promising. Mutations can occur in two ways: spontaneous mutagenesis and induced mutagenesis (chemical mutagenesis, UV Radiation mutagenesis, and ionizing radiation) [1]. In the late 19th century, Hugo de Vries identified mutations as a means of generating variability while researching on Mendel’s laws of inheritance [2]. Mutations, the heritable changes in the genome are the driving force for the evolution of any organism [3]. By means of inducing mutation using different mutagenesis approaches, evolution can be accelerated or directed to achieve the desired change in an organism. Induced mutagenesis has been widely used for the genetic improvement of all the organisms having human interest including microbes, animals, and plants. In plant breeding programs, physical and chemical mutagens are successfully applied for the development of new varieties with enhanced traits [4].

Mutagenesis is also a promising tool to study a biological system. In plant biology research, different mutagenesis approaches have been used to identify novel genes and their functional regulations. The most commonly used mutagenesis approaches include chemicals, ionizing radiation, and T-DNA insertion [5]. Insertional mutagenesis is a method that disrupts the DNA sequence, allows the rapid identification and isolation of the gene. It can be mutated through T-DNA insertion or transposons while each having their pros and cons [6]. Chemical and ionizing radiation mutagenesis, are although random approaches, but are relatively more straightforward and manageable [7]. Ethyl methane sulfonate (EMS) is the most commonly used alkylating agent, for creating a mutagenized population [5]. The chemical mutagenesis approaches are the most affordable options but impose great risk to the researcher as well as the environmental health. In contrast, ionizing radiation mutagenesis needs expensive instruments and specialized laboratory setup. Moreover, the mutations caused by the chemical mutagens are genetically less stable; there are chances that the mutations revert to the original wild-type form. Recently, zinc finger nucleases (ZFNs), transcription activator-like effector nucleases (TALENS), and clustered regularly interspaced short palindromic repeats (CRISPR)/CRISPR-associated9 (Cas9) endonuclease were popularly used to target the gene of interest to increase the productivity in crop plants [8].

Each mutagenic approach generates a specific type of mutation. For instance, RNA induced gene silencing usually creates the gene expression knockdown, resulting in the gene function silencing [9,10]. Similarly, the T-DNA or transposon insertion disturbs the codon reading frame resulting in a deformed protein or transcript [11]. Whereas, the EMS treatment to seeds creates a high frequency of point mutation in DNA [4,9]. Advantages and disadvantages of different mutagenic approach are mentioned in Table 1.

The ionizing radiation (IR) of both nonparticulate (e.g., gamma and X-rays) and particulate (neutron, heavy ions) impacts relatively varying size of deletions ranging from few bases to few million bases (Mb) [12]. The type of DNA damage caused by various ionizing radiation depends upon the relative biological effectiveness (RBE) of the radiation, which in terms is the function of its linear energy transfer (LET) [13]. Gamma rays are the most commonly used ionizing radiation source in mutation breeding program. However, with development of high-energy cyclotrons and research reactors particulate IR like, heavy ion and neutrons (fast and thermal) with many fold high LET compared to gamma rays have become available for use in mutagenizing experiments for mutation breeding. Gamma rays being low in LET radiation, there is increasing trend toward use of high LET particulate radiation source for mutation induction; moreover, the large deletions created with gamma rays reported are usually not inherited stably [14].

Fast neutron mutagenesis is relatively a less explored approach, usually creates large deletion (sometimes >1 Mb), and also results in the chromosome rearrangement in the genome [15]. Fast neutron mutagenesis uses fast neutron irradiation or neutron bombardment to generate a mutagenized population. Fast neutrons have been reported to cause higher number of non-repairable double lesions and also the repair of DNA lesion is highly delayed compared to gamma or X-rays [18]. In addition, when compared to gamma rays, fast neutron has higher frequency of double strand break (dsb), and hence higher RBE. Fast neutron has been found to be a very efficient mutagen in plants, and it is easy to generate the FN-treated lines, and rapid assembly of deletion library aids to find deletion mutant [19]. It is one of the approaches that is found to be highly efficient in generating a library of gene knockouts in plants. FN mutagenesis has been successfully reported in many species, including *Arabidopsis* [20], soybean [21], rice [17], *Medicago truncatula* [22], peanut [23], and *Lotus japonicas* [24]. The recent increase in the use of fast neutron irradiation as a mutagen is due to the technological advances in mutagenesis treatment as well as the next-generation sequencing-based mutation mapping approaches [14,21]. The size of the deletion ranges from 1 bp to 18 Mb, while most results in 1 to 4 kb deletion [17]. However, Belfield et al. [20] reported that fast neutron generates single base substitution with a higher frequency as compared to large deletions. They also found that transitions were occurring at a higher rate at pyrimidine dinucleotide sites implying the establishment of covalent linkage due to mutation between adjoining pyrimidine residues. Knockout mutants induced through fast neutron or gamma rays are beneficial for tandemly repeated gene families [9]. In many plant species, it has been used to create a mutagenized population [17].

## 2. Fast Neutron Radiation-Induced Mutagenesis

**Figure 1:**
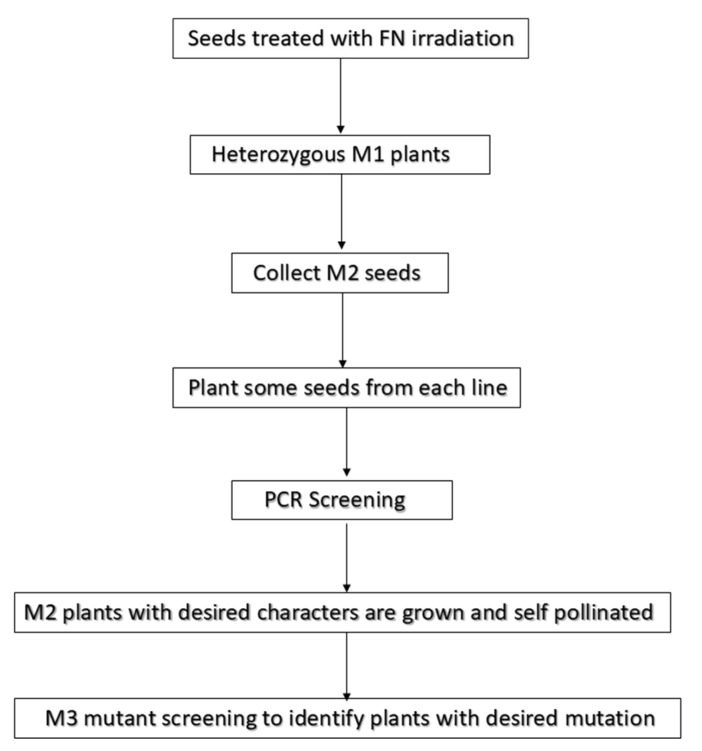
Generalized flow chart showing steps involved in the development and evaluation of mutant population derived from the plants treated with fast neutron.

Fast neutron radiation is found to be a potent mutagen in plants [25]. A seminal study performed by James Chadwick in 1932 discovered neutrons as small particles of matter having no electric charge [26,27]. Since neutrons do not carry any charge, they do not ionize directly but occur as a structural unit of atomic nuclei of particles heavier than hydrogen. When atomic nuclei encountered a specific type of disintegration, high energetic neutrons are released known as fast neutrons or neutron rays. Since these neutrons are neutral and non-ionizing, which accounts for their ability to penetrate in-depth of the matter. Fast neutrons beam of adequate intensity for the biological tissue were made available by the discovery of cyclotron by E. O. Lawrence [27]. It causes ionization indirectly through the collision of nuclei of atoms, mainly hydrogen, in the tissue [28]. However, the RBE of FN in biological tissue is distinctly contributed by two interactions, first the interaction of neutron with proton (H^+^) and second from interaction with other heavy nucleus [29]. Fast neutrons cause high proportions of irreparable dsb in genome, which leads to mutations [30]. Earlier, fast neutron mutagenesis was used to study forward genetics in plants, but now it has also been applied in reverse genetics studies [14]. To build a link between the biochemical role of a gene or to reveal a biochemical pathway the in vivo reverse genetics approaches are proven efficient. In reverse genetics, the central technique currently implemented for fast neutron mutagenesis is Deletagene (delete-a-gene) [15]. In this technique, fast neutron mutagenesis is combined with high throughput PCR screening to achieve deletion mutants of targeted genes (Figure 1). The Deletagene approach was first demonstrated in *Arabidopsis* a model plant species and rice [14]. During fast neutron irradiation, the important thing is to determine the appropriate dose (lethal dose 50 or LD50) of radiation for high mutagenic efficiency and characteristics of the tissue that is being exposed to these radiations to obtain optimal seeds for further studies [14,31]. If sufficient data is available in the literature about dose treatment, then that can be used efficiently to plan appropriate experiments. For example, 60 Gy (Gray, 1 Gray = 100 radiations) dosage level; is used in *Arabidopsis thaliana*, 4–32 Gy in Soybean, 20 Gy in rice, and 30–40 Gy in *Medicago truncatula* [17,20,21,22]. Species in which data is not available on optimal dosage, pilot experiments need to be conducted in which a small number of seeds are treated with different dosage, to determine the optimal dosage. A 50% survival of M1 treated plants is usually considered as an adequate balance between mutagenesis and fertility. But the dose can be higher or lower considering the required frequency of mutations. Mutation rate can also be determined by segregating albino phenotypes in the M2 progeny [32]. In *Arabidopsis*, 2% albino frequency has been estimated when mutagenized with 60 Gy fast neutron [14,25]. RBE studies of FN irradiation were studied in different crop species. These studies estimate RBE of FN over gamma rays ranging from 3–13 for different end points like germination percentage, fertility, seedling length, chlorophyll mutants, and chromosomal aberrations. In one such study single locus mutation was examined in einkorn wheat, and it was found that FN (14 MeV) had 13 times higher RBE compared to gamma rays [33]. Another convenient option is the use of PCR to amplify the selected genomic region or random regions [34]. PCR screening can be done in 2D or 3D grids depending on population size and PCR detection limits [19]. However, not all mutations are detectable through PCR screening, only deletions greater than 500 bp are detectable. Therefore, mutations induced by FN that are mostly large deletions are more likely to be detected with the PCR approach. Apart from getting a glimpse of mutation frequency the PCR approach can also be used for the mapping of causal mutation (discussed in sections below). Fast neutron mutagenesis is implemented in diverse organisms as mentioned in Table 2.

## 3. Combining FN Mutagenesis with Other Mutagenesis Methods

Mehandjiev, et al. [46] combined different chemical mutagens and ionizing irradiation to create a huge effect on the genetic structure. They first treated pea seeds with FN or gamma rays and then with the chemical mutagen. Pronounced results were obtained from a combination of 10 Gy FN and 0.2% EMS in which a hyper-additive effect was also seen. These effect produced higher genetic variability and also enhanced the chances for screening mutants containing the desired trait. For a better understanding of oxidative stress and mutagenic potential a study was conducted in which various irradiation doses of UV-B and FN were produced, generating various levels of ROS on the pollen mother cell and seed yield of *Vicia faba* seedling (Table 3) [47].

## 4. Genetic Stability of Mutants Generated Using Fast Neutron Radiations

FN mutagenesis results in genetic variations, deletions, and translocations. The estimation of DNA damage and study of the stability of mutants is imperative to utilize these mutants in selection for desired phenotypes and deduction of genes of interest [49]. Primarily, the stability of FN mutants was assessed only in M1 and M2 generations. A study was undertaken to check the stability of M3 FN mutants in *Triticum aestivum* and a decrease was observed in pollen sterility due to persistent chromosomal aberrations [50]. Most of the limited studies related to the elucidation of genetic stability of FN mutants have been conducted in the previous decade and only a few of these were based on crop plants. For instance, a study was conducted to estimate the effect of FN mutagenesis on 264 soybean crop plants (*Glycine max*) that showed higher levels of segmental duplications in its genome when compared with previous studies [49]. Similarly, analysis of genetic stability of mutants in non-crop plants such as *Arabidopsis thaliana* for 20 alleles of *Hy4* locus was performed and it was observed that while nine mutants had stable genetic behavior, remaining mutant alleles were lethal in homozygous conditions [51]. Another study analyzed *Arabidopsis thaliana* FN-induced mutants and the results suggested the formation of covalent bonds between adjacent pyrimidine bases [20]. As evident, although FN mutagenesis along with other physical and chemical mutagenesis techniques has been utilized for gene discovery, detailed studies related to genetic stability of FN mutants in crop plants are limited in number. 

## 5. Fast Neutron Mutagenesis in Polyploidy Plant Species 

Polyploidy is common in plant species such as, durum wheat, cotton, tobacco, peanut (tetraploid), bread wheat and kiwifruit (hexaploid), dahlias and sugarcane (octoploid), and strawberries (decaploid). Ploidy levels along with ionization density affect the response of plant species when subjected to radiations like FN [52]. Due to polyploid nature these genomes are highly tolerant to mutation; for example in case of a hexaploid wheat, the mutation rate is reported to be 1 per 32 kb compared to 1 per 400 kb in diploid crops [53]. As a result all copies of the gene must be mutated in the polypoid genome (e.g., six in case of hexaploid wheat) to express the desired mutation. It was generally hypothesized that as compared to diploids, polyploids must be affected less by radiation-induced mutagenesis due to the presence of duplicated sets of genes which led to detailed studies in crop plants like wheat, cotton, and barley [54]. Mutagenesis is tricky in polyploidy species owing to the difficulty in defining the target region [55]. One such study that researched the relationship between polyploidy and FN radiations among others was conducted and subsequently the effect of radiations was employed as a tool to evaluate level of ploidy of *Hordeum vulgare* (Barley), *Triticum aestivum* (wheat), *Nicotiana rustica* (tobacco), and *Gossypium hirsutum* (cotton) [55]. Studies have been undertaken to explore comparative effects of FN mutagenesis on plants with different levels of ploidy. Effect of FN mutagenesis has been comparatively studied in tobacco and rice, wherein morphological changes have observed in tobacco leaves and stem [56]. FN mutagenesis in important polyploidy crop plants has also been used to identify genetic regions responsible for susceptibility or resistance to diseases. For instance, a deletion induced by FN irradiation in chromosome arm 3BS of wheat landrace *Wangshuibai* increased crop susceptibility to wheat scab or *Fusarium* head blight [57]. Similar studies have been conducted to explore the effect of FN mutagenesis on stress tolerance and yield of staple crops like wheat, for example. FN irradiation in wheat cultivar (Sakha 92) for three successive seasons resulted in increased yield and higher tolerance to salt stress, in addition to increase in sugar concentrations and crude protein [58]. Although FN mutagenesis has immense application in crop plants, most of the studies utilizing this method have been conducted in polyploidy crop plant species like wheat as compared to other plants. 

## 6. Mapping of Mutation Induced by Fast Neutron Radiations

The significant advantage of fast neutron-based mutation is that they are effortless to create and map, particularly with the next-generation sequencing (NGS) techniques. The mapping of casual mutation helps to explore the forward or reverse genetics applications. The availability of the high throughput sequencing technologies has provided enormous information about genomic and transcriptomic sequences of numerous crop species, yet the functions of most of the genes are unknown. The conventional gene silencing strategies like, RNAi and intron splicing are only partially effective and involve the highly time-consuming transgenic plant creation. On the other hand, another mechanism of T-DNA insertion is comparatively rapid in PCR-based screening procedures, but mostly the insertion of a transposable element in the regulatory or the coding regions of the gene led to the disruption in its function. A large number of insertional mutant libraries have been reported in many crop species, but it is challenging to create insertional mutants for the whole genome of a crop plant. Another concern is that the commercial use of only non-transgenic mutated alleles is possible. In this regard, FN mutagenesis is a promising way to develop a mutant population and subsequently perform the mapping for causal mutations. Based on NGS, several mutation mapping approaches including, mapping by mutation (MutMap), MutMap-Gap, mutant chromosome sequencing (MutChromSeq), exon capture, tilling, Mut-Ren-Seq have been developed and are being efficiently explored. Similarly, the whole genome sequencing and genotyping by sequencing are also convenient options for pinpointing causal mutations.

## 7. PCR-Based Candidate Gene Screening for the Localization of Mutations

As discussed in above sections, the FN approach usually creates larger deletions that can be easily detected by PCR amplification of target site in the genome. In brief, after getting the M2 mutant lines that are segregating for the mutations, phenotyping can be performed to identify mutants with desired characters. Then the candidate genes for the target traits can be identified based on the genomic information from the same or related species. The selected candidate genes can be amplified using specific primer in mutant and the parental wild types and then the amplicons size need to be compared on agarose gel electrophoresis [20]. The PCR-based screening can be more cost and time efficient if performed with pooling large number of lines for the first round of screening. Once the pool is confirmed to have the deletion then in second step each mutant from the pool can be screened to confirm the deletion in the particular mutant [14,19,34].

## 8. MutMap: An Efficient Approach for Mutation Mapping in Small Genomes

**Figure 2:**
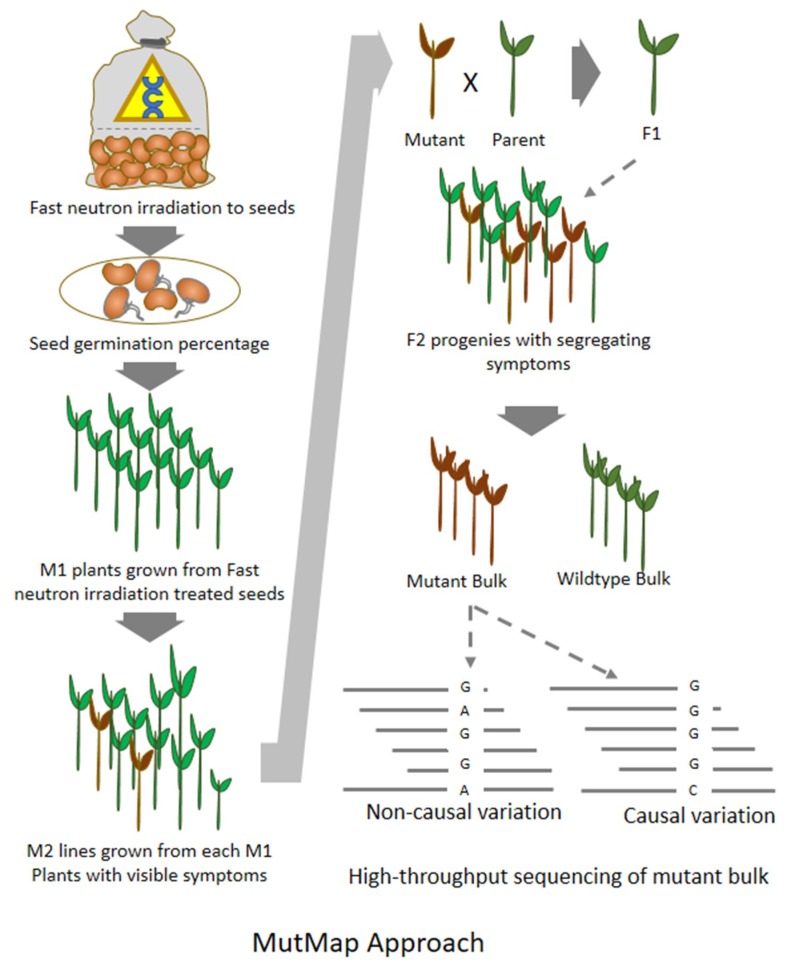
Generalized flowchart of mapping by mutation (MutMap) approach being used for the localization of casual mutation in small plant genomes like rice.

Mapping by mutation (MutMap) is a forward genetics method devised to accelerate the process of crop breeding and rapid isolation of genes over QTL mapping (Quantitative Trait Loci) or gene cloning. The principle behind MutMap is the identification of mutant phenotype by whole genome sequencing and tracing it back to the reference genome by SNPs (Single Nucleotide Polymorphism) indexing. The MutMap approach is based on the SNP identification and subsequently retains only those SNP that are linked to the mutant phenotype. In this methodology, a mutation is created in a cultivar using a mutagen and then it is selfed to produce the seeds for F2 generation. In the F2 generation, all the mutants are screened for the desired phenotypes. The mutant plants with the desired phenotype are backcrossed with their wild type parental cultivar to produce F1 plants, which need to be selfed to produce the F2 generation. Based on the phenotypic evaluation of F2 progenies, two contrasting bulks representing wild type phenotype and mutant phenotype are created. Then the DNA sample of the mutant bulk need to be sequenced and aligned to the available reference genome to identify SNPs and InDels (Figure 2). The SNPs unanimously present in all the reads from mutant bulk therefore represent the association with mutant phenotype and these are the most probable causal mutations, rest of the segregating SNPs are the non-associated mutations [59]. Using the MutMap approach a study was carried out in *M. trunculata* to find out the salt-tolerant genes. In this study, they exposed wild type *M. truncatula* (ecotype Jemalong A17) seeds to the fast neutron rays at the 35 Gy (Gray, 1 Gray = 100 radiations) dosage level. The seeds were planted, selfed, and selected for the desired phenotype, and those selected plants were backcrossed with the parental line. Subsequently, causal mutations in two genes associated with the salt stress phenotype have been identified using the MutMap approach [60]. Many genes having significant role in agronomically important traits in rice, tomato, soybean have been identified using the MutMap approach. Most of such studies exploring the MutMap have used EMS. Very limited effort has been devoted for the use of MutMap in FN-based mutant population in crop plants. MutMap approach has some limitations where reference genome is not available or when the genome is several giga bases in size with higher ploidy level. However, for the true diploid plants with less than 1 GB genome size, MutMap is a promising method.

**Figure 3:**
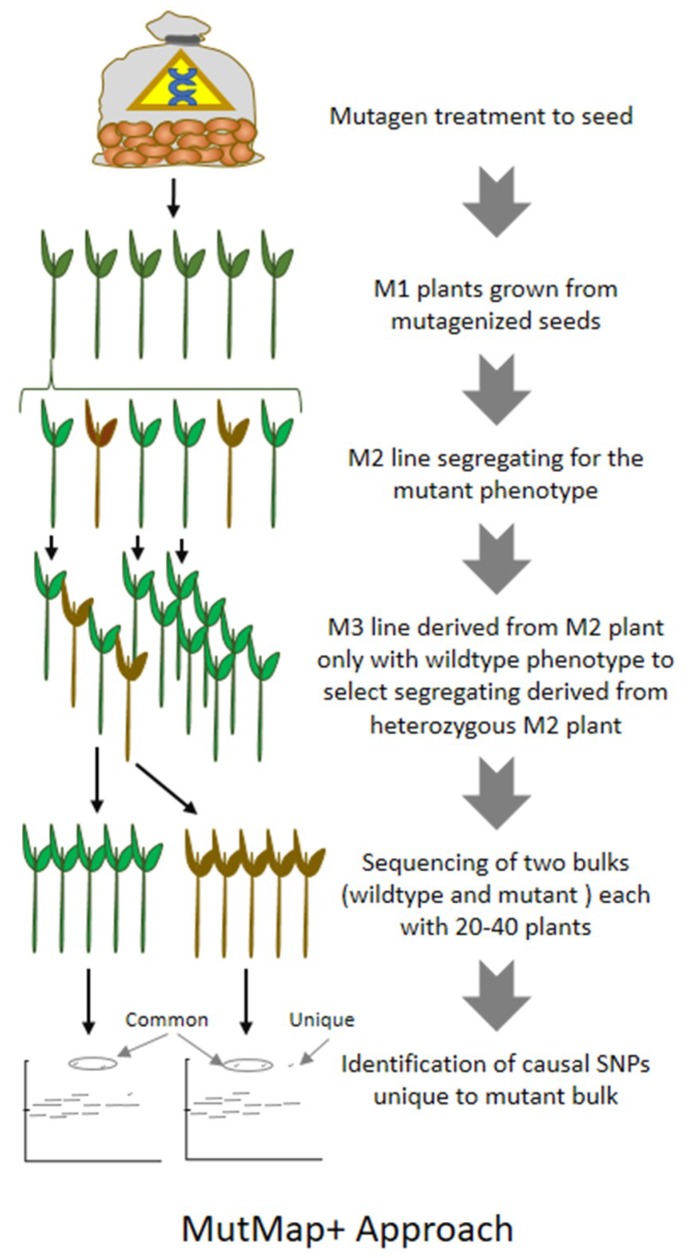
Schematic representation of MutMap+ strategy exploring next-generation sequencing for identification of casual SNP unique to bulk mutant.

Efforts have also been made to further simplify the MutMap approach by making bulk of mutant plants and wild type plants directly from the segregating lines instead of crossing mutant to parental line (Figure 3). Such improved or simplified approach is termed as MutMap [60]. The first report of MutMap+ where DNA from mutant and wild type plants from M3 segregating line derived from M2 were bulked and sequenced to identify causal mutations in rice. The approach saves considerable efforts and time as compared to MutMap. The approach is more promising where artificial crossing is challenging and also for plants that take longer time for the generation advancement. 

## 9. MutMap-Gap

**Figure 4:**
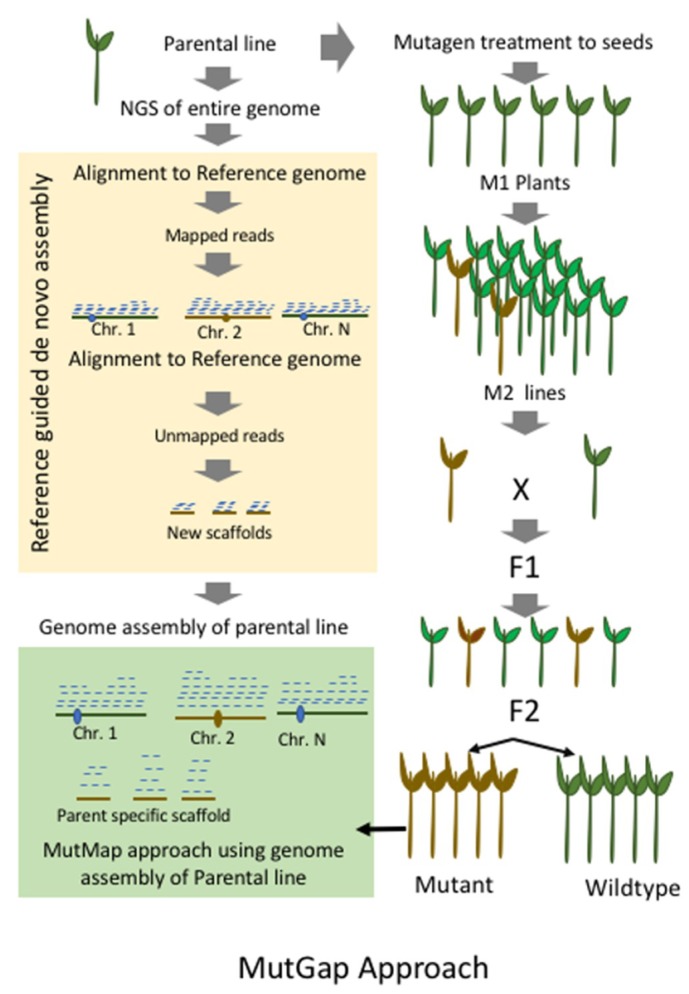
Generalized flowchart of MutMap-Gap approach in which reference parental genome created and gap regions in the parental/cultivar genome identified. Subsequently, MutMap approach needs to be followed as described in Figure 2.

MutMap-Gap is the specialized version of MutMap technique based on the principles of targeted assembly of gap regions in the genomic DNA and mapping by mutation (Figure 4). The major disadvantage of the MutMap methodology is the mandatory presence of the genomic sequence flanking the mutation in the reference parental genome. The reference parental genome of a cultivar/line (understudy) is created by replacing all the known SNPs of the reference genome of that crop species. When the parental line/cultivar shows some of the significant structural differences with the reference genome, then the mutation sites are difficult to map by alignment methods. MutMap-Gap is a methodology to expand the horizons of MutMap by sequencing techniques, and the gap regions in the targeted mutation are reconstructed by the de novo assembly of the reference sequences (Figure 4). A blast resistance gene *Pii* was isolated in a rice cultivar *Hitombore* based on loss of function in a mutant line by MutMap-Gap technique [61]. No reports of MutMap-Gap technique using fast neutrons as the mutating agent have been published so far. 

## 10. MutChromSeq: A Method Based on Selected Chromosome Sequencing 

MutChromSeq or mutant chromosome sequencing is a recently discovered technique to locate mutation in polyploid and large complex genomes. Whole genome sequencing of the mutant plants with the polyploidy or large genome is costly, laborious, and computationally challenging. This technique combines the applications of the classical mutagenesis with the recent advances in the field chromosome flow sorting and sequencing. The prerequisite for this technique is that the cultivar/line under study should be responsive to mutagenesis, the phenotype for the desired mutation should be significant, and the information regarding the position of the gene on the chromosome should be known by default. The mutants are created, selfed, and selected for the desired phenotype in the M_3_ generation classically and the suspensions of selected plant sample are prepared to label the mitotic chromosomes fluorescently with the help of labeled DNA-based probes. The tagged chromosomes are then sorted on the basis of fluorochrome signals. Sequencing of mutant and wild type chromosomes are performed for the gene identification [62]. 

## 11. Exon Capture

Exon capture or exon resequencing is based only on the protein coding region of the genome. It comprises only 1 to 2% of the whole genome. The most significant advantage of relying on this technique is that because of the small sample size, large number of samples can be analyzed to identify the desired traits. The first phase of this approach includes target enrichment which includes hybridizing the sequences in DNA that codes for a protein with the help of probes. The next phase includes high throughput DNA sequencing. Exon capture is a better approach than RNA sequencing as it is not dependent upon tissue, stage, or transcript abundance, but upon genes and alleles [63]. A study was conducted on soybean to create a public resource to be used in functional genetic researches as genetic screens. They conducted their experiments using FN beam at different dosage levels (4, 8, 16, 32 GY) to induce the mutations, and analyzed their results via exon capture and exon resequencing [21]. 

## 12. MutRen-Seq

One of the specialized extensions of exome capture method has been its use in the identification and cloning of disease resistance gene (*R* gene) using MutRen-Seq. MutRen-Seq combines mutagenesis with exome capture and then sequences for rapid identification and cloning of R genes. Although the original method employs chemical mutagenesis, it is equally applicable for physical mutagens like FN. The approach functions by creating a loss of function mutations for R gene in plants. Genomic library from these mutants and parents were enriched for R gene coding regions using 120-mer RNA probes/baits designed from homologous R gene sequences from many related species. This enriched library is then sequenced and putative causal mutated genes identified, which can be further cloned using the bait sequence. This method has been demonstrated in wheat for cloning of stem rust resistance gene *Sr22* and *Sr45* [62]. 

## 13. Targeting Induced Local Lesions in Genomes (TILLING) Approaches

Targeting induced local lesions in genomes (TILLING) is a widely accepted technique to identify a gene of interest in any crop genome [64]. The TILLING approach is one of the most efficient tools for reverse genetics, which is based on conventional mutagenesis and an NGS-based screening procedures to identify desired mutations in the trait of interest. This process involves the induction of mutation and development of F2 generation by selfing. The DNA sample is prepared and PCR is carried out in the gene of interest with the help of fluorescently labeled primers. The heteroduplexes formed at the site of mutation are then digested with restriction enzymes and analyzed on polyacrylamide denaturing gel. Different studies were carried out using the applications of TILLING in a variety of crop species. If TILLING approach needs to be used for the FN-induced mutations, then significant modification is required to identify simultaneously the SNPs, small InDels, and large deletions.

## 14. Whole Genome Sequencing

Whole genome sequencing is comparatively a costly option for the mutation detection but provides in-depth understanding of the genome-wide variations caused. In case of SNPs and InDels, cause of the variation is difficult to confirm, since similar variations can spontaneously have occurred in the genome with advancement of every generation. However, larger deletions and chromosomal rearrangements as usually observed with the FN mutagenesis have very less chances to occur spontaneously. The whole genome sequencing of fast-neutron-mediated mutant lines provides opportunity to develop a genomic resource that can be used for forward and reverse genetic applications [65]. One of the excellent example where FN mutants have been sequenced to develop a genomic resource is the study performed on soybean [21]. The soybean FN mutant’s whole genome sequence data is widely being used to identify novels gene. Similar efforts are expected for many other important crop plants. 

As NGS-based whole genome sequencing generates huge data sets, which needs to be compared and correlated for identifying casual mutations, many software tools and pipelines have been developed for analyzing NGS data for mutation mapping e.g., SNPtrack, SHOREmap, NGM, and SIMPLE. SIMPLE is one of the user friendly tools designed that can be used with no knowledge of programming language and very little pre-preparation data [66].

## 15. Genotyping by Sequencing-Based Mutation Mapping

Genotyping by sequencing (GBS) is a technique that broadens the horizon of next-generation sequencing technologies by combining the applications of molecular markers and sequencing technologies. It is used for crop breeding in crops with complex genotypes. This methodology depends on the digestion of the genomic DNA samples with the restriction enzymes. The adapters are ligated to the fragments and are then amplified with the help of PCR specific primers and libraries are prepared. These libraries are then sequenced and the sequenced reads are then aligned to the reference genome for SNP indexing. It has been an excellent marker-assisted tool that can be efficiently and cost effectively used in crop breeding practices [67]. A study based on identifying spontaneous mutation induced by FNB for broad spectrum resistance against brown planthopper was studied. They used double digest restriction site-associated DNA sequencing (ddRADseq) technique for identification of brown plant hopper resistance genes in rice [68]. The GBS approach will be also helpful to identify the large deletion and create a genomic recourse similar to the whole genome sequencing approach, but with many fold lower cost. 

## 16. Challenges for the Efficient Utilization of FN Mutagenesis Approaches for Crop Improvement 

Mutagenesis aims at targeted genetic variations while minimizing the effect on the viability of germplasm. Despite immense application in gene discovery and isolation, FN mutagenesis is faced with multiple challenges. FN mutagenesis results in large deletions in genetic regions, in addition to causing translocations and chromosomal loss [69]. Moreover, novel mutations in regulatory or coding regions may disrupt molecular pathways, which are not extensively studied yet [70]. Large deletions and translocations pose a challenge due to the lower resolution of variation as compared with other mutagenesis approaches viz. chemical mutagenesis, which offer more precise single nucleotide polymorphisms [69]. Based on the dose of irradiation and response of plant species toward FN mutagenesis, varying degrees of genetic variations are observed. In spite of the presence of antioxidant scavenging mechanisms, FN mutagenesis induces production of reactive oxygen species (ROS) resulting in unchecked oxidation in cells [47]. In addition, certain plant crops have longer generation times that impede the fast and time-saving approach adopted by FN mutagenesis [17]. The FN mutagenesis demands a higher focus on avoiding injury to plant cellular compartments with varying dosage [71]. Moreover, identification of larger genomic deletions that are laborious to be identified by conventional methods requires a large sample size even with FN mutagenesis [72]. Also, utilization of FN mutagenesis in case of reverse genetics techniques for polyploidy plant species such as, wheat, poses challenges due to high similarity amongst homoeologous loci [53]. Multiple complex chromosomal rearrangements result from FN mutagenesis and impede gene identification and isolation in crop plants [73]. Several strategies have been suggested to minimize the disadvantages of FN mutagenesis viz. traditional backcrossing assists in removing non-specific variations [20]. In spite of the aforementioned challenges, FN mutagenesis, when combined with high throughput methods such as next-generation sequencing (NGS) technologies, proves to be an indispensable tool for faster gene identification and isolation.

## 17. General Conclusions 

Extensive studies have been conducted in the past few decades to explore nucleotide diversity of genomes which are of utmost importance to humans. Among these, the most studied genomes belong to crop plants. Owing to limited availability of natural variations, which can be exploited for further improvement in genetic diversity, various techniques have been devised to further the cause. Fast neutron mutagenesis is one of the most important methods among various other physical and chemical mutagenesis techniques. FN mutagenesis supersedes preceding methods in terms of better safety, rapid and efficient utilization in plants. Although extensive studies are still needed to measure the stability of induced mutations, efficient dosage, and exposure in a wide array of polyploidy species, it is one of the fore runners in emerging mutagenesis studies. FN mutagenesis is highly amenable to pairing with other mutagenesis, screening (PCR-based methods), and mapping techniques (NGS techniques), and hence can be further explored to realize its full mutagenesis potential. 

## Figures and Tables

**Table 1:** Advantages and disadvantages of different mutagenic approaches.

Method	Advantages	Disadvantages	References
Chemical mutagenesis	Identification of multiple alleles of genes. Heritable.	Isolation of mutated gene is difficult	[6,15]
Insertional mutagenesis	Chemically and physically Stable through multiple generations. T-DNA can be used to study specific stages of cellular differentiation or cell fate.	Transactivation properties. Require transformation	[6,11,16]
Fast neutron mutagenesis	Saturate the genome. Do not require transformation or tissue culture. Complete knockout of gene	Screening is time taking. Can delete multiple genes at a time	[15,17]

**Table 2:** Study of fast neutron mutagenesis in different organisms.

Organism	Objective	References
Mouse	Effect of chronic neutron- and γ-irradiation on spermatogonia	[35]
Methanogenic bacteria (TDM, TRM, and SSM)	Effect of physical irradiation (y irradiation, neutron bombardment) and chemical mutagen (acridine orange and colchicine) on methane production	[36]
*Drosophila melanogaster*	Induced rates of mitotic crossing-over	[37]
Yeast	Relative biological effectiveness of 14.5- MeV neutrons for the induction of gene conversion	[38]
*Musca domestica L*	To compare effect of fast neutrons and gamma rays in producing sterility	[39]
*Melanoplus differentialis*	To study fast neutrons, gamma rays, X-rays relative biological effectiveness on nymph ovarioles	[40]
*Tribolium Castaneum*	Effect of fast neutron on productivity of young and old flour beetles	[41]
*Trichoderma viride*	Effect of FN on production of cellulose	[42]
Chinese hamster	Response of ovary cells to fast neutron	[43]
Mammalian cells	To study mutations induced by γ-rays and fast neutrons	[44]
*Chlorella* sp.	Enhancement of lipid productivity	[45]

**Table 3:** Details of significant studies performed with fast neutron (FN) radiations and FN combined with other mutagens.

Species	Mutagen	Dose	Number of Mutants	Reason	References
Soybean cv M92-220	FN	4–32 Gray units	23,000	Phenotypic screening and associated genomic characterization	[21]
*Arabidopsis thaliana*	FN	60 Gy	300	Screening of elongated hypocotyl mutants	[20]
Rice	FN	20 Gy	2418	For genome-wide profiling of mutations	[17]
Peanut	FN + in vitro culture	9.7–18.0 Gy	19	Somatic embryogenesis combined with plant regeneration	[23]
*Medicago truncatula*	Fast neutron bombardment (FNB)	30–40 Gy	1000	creened for symbiotic nitrogen fixationSymbiotic nitrogen fixation mutant lines	[22]
*Vicia faba* L.	Fast neutron (FN) and UV-B	280–320 nm 30–40 Gy		To study combined effect of oxidative stress and mutagenic potential	[47]
*Lotus japonicas*	FN	8 Grey (Gy)	58	Non-nodulation mutant called FNN5-2	[24]
*Lycopersicon esculentum* (M82)	FN	15 Gy	865	Functional genomic studies	[48]
*Pisum sativum*	FN + EMS	10 Gy + 0.2% EMS	14%	Mutation genetics and breeding study	Mehandjiev, Kosturkova and Mihov [46]

## References

[1] Friedberg E.C., Walker G.C., Siede W., Wood R.D. (2005). DNA Repair Mutagenesis.

[2] Oladosu Y., Rafii M.Y., Abdullah N., Hussin G., Ramli A., Rahim H.A., Miah G., Usman M. (2016). Principle and application of plant mutagenesis in crop improvement: A review. Biotechnol. Biotechnol. Equip..

[3] Ripley L.S., Maloy S., Hughes K. (2013). Mutation. Brenner’s Encyclopedia of Genetics.

[4] Kodym A., Afza R. (2003). Physical and chemical mutagenesis. Plant Functional Genomics.

[5] Tadege M., Wang T.L., Wen J., Ratet P., Mysore K.S. (2009). Mutagenesis and beyond! Tools for understanding legume biology. Plant Physiol..

[6] Topping J.F., Lindsey K. (1995). Insertional mutagenesis and promoter trapping in plants for the isolation of genes and the study of development. Transgenic Res..

[7] Wu J.-L., Wu C., Lei C., Baraoidan M., Bordeos A., Madamba M.R.S., Ramos-Pamplona M., Mauleon R., Portugal A., Ulat V.J. (2005). Chemical-and irradiation-induced mutants of indica rice IR64 for forward and reverse genetics. Plant Mol. Biol..

[8] Chaudhary J., Alisha A., Bhatt V., Chandanshive S., Kumar N., Mir Z., Kumar A., Yadav S.K., Shivaraj S., Sonah H. (2019). Mutation Breeding in Tomato: Advances, Applicability and Challenges. Plants.

[9] Tadege M., Ratet P., Mysore K.S. (2005). Insertional mutagenesis: A Swiss Army knife for functional genomics of Medicago truncatula. Trends Plant Sci..

[10] Hebert C.G., Valdes J.J., Bentley W.E. (2008). Beyond silencing—Engineering applications of RNA interference and antisense technology for altering cellular phenotype. Curr. Opin. Biotechnol..

[11] Krysan P.J., Young J.C., Sussman M.R. (1999). T-DNA as an Insertional Mutagen in Arabidopsis. Plant Cell.

[12] Sakai K., Suzuki S., Nakamura N., Okada S. (1987). Induction and subsequent repair of DNA damage by fast neutrons in cultured mammalian cells. Radiat. Res..

[13] Bewley D. (1968). A comparison of the response of mammalian cells to fast neutrons and charged particle beams. Radiat. Res..

[14] Li X., Zhang Y. (2002). Reverse genetics by fast neutron mutagenesis in higher plants. Funct. Integr. Genom..

[15] Gilchrist E., Haughn G. (2010). Reverse genetics techniques: Engineering loss and gain of gene function in plants. Brief. Funct. Genom..

[16] Alonso J.M., Stepanova A.N., Leisse T.J., Kim C.J., Chen H., Shinn P., Stevenson D.K., Zimmerman J., Barajas P., Cheuk R. (2003). Genome-Wide Insertional Mutagenesis of *Arabidopsis thaliana*. Science.

[17] Li G., Chern M., Jain R., Martin J.A., Schackwitz W.S., Jiang L., Vega-Sánchez M.E., Lipzen A.M., Barry K.W., Schmutz J. (2016). Genome-wide sequencing of 41 rice (*Oryza sativa* L.) mutated lines reveals diverse mutations induced by fast-neutron irradiation. Mol. Plant.

[18] Hendry J.H. (1991). The slower cellular recovery after higher-LET irradiations, including neutrons, focuses on the quality of DNA breaks. Radiat. Res..

[19] Li X., Song Y., Century K., Straight S., Ronald P., Dong X., Lassner M., Zhang Y. (2001). A fast neutron deletion mutagenesis-based reverse genetics system for plants. Plant J..

[20] Belfield E.J., Gan X., Mithani A., Brown C., Jiang C., Franklin K., Alvey E., Wibowo A., Jung M., Bailey K. (2012). Genome-wide analysis of mutations in mutant lineages selected following fast-neutron irradiation mutagenesis of *Arabidopsis thaliana*. Genome Res..

[21] Bolon Y.-T., Haun W.J., Xu W.W., Grant D., Stacey M.G., Nelson R.T., Gerhardt D.J., Jeddeloh J.A., Stacey G., Muehlbauer G.J. (2011). Phenotypic and genomic analyses of a fast neutron mutant population resource in soybean. Plant Physiol..

[22] Chen Y., Chen R. (2018). Physical Mutagenesis in Medicago truncatula Using Fast Neutron Bombardment (FNB) for Symbiosis and Developmental Biology Studies. Functional Genomics in Medicago Truncatula.

[23] Wang J.-S., Sui J.-M., Xie Y.-D., Guo H.-J., Qiao L.-X., Zhao L.-L., Yu S.-L., Liu L.-X. (2015). Generation of peanut mutants by fast neutron irradiation combined with in vitro culture. J. Radiat. Res..

[24] Hoffmann D., Jiang Q., Men A., Kinkema M., Gresshoff P.M. (2007). Nodulation deficiency caused by fast neutron mutagenesis of the model legume *Lotus japonicus*. J. Plant Physiol..

[25] Koornneeff M., Dellaert L., Van der Veen J. (1982). EMS-and relation-induced mutation frequencies at individual loci in *Arabidopsis thaliana* (L.) Heynh. Mutat. Res. Fundam. Mol. Mech. Mutagenesis.

[26] Goodhead D.T. (2019). Neutrons are forever! Historical perspectives. Int. J. Radiat. Biol..

[27] Aebersold P.C., Lawrence J.H. (1942). The physiological effects of neutron rays. Annu. Rev. Physiol..

[28] Smith H.H. (1958). Radiation in the production of useful mutations. Bot. Rev..

[29] Dunning J., Pegram G., Fink G., Mitchell D. (1935). Interaction of neutrons with matter. Phys. Rev..

[30] Tanaka K., Gajendiran N., Endo S., Komatsu K., Hoshi M., Kamada N. (1999). Neutron energy-dependent initial DNA damage and chromosomal exchange. J. Radiat. Res..

[31] Brunner H. (1995). Radiation induced mutations for plant selection. Appl. Radiat. Isot..

[32] Rogers C., Wen J., Chen R., Oldroyd G. (2009). Deletion-based reverse genetics in Medicago truncatula. Plant Physiol..

[33] Fujii T. (1964). Relative Biological Effectiveness of 14-MeV Fast Neutrons to Co 60 Gamma-Rays in Einkorn Wheat. Biological Effects of Neutron and Proton Irradiations. Vol. II. Proceedings of the Symposium on Biological Effects of Neutron Irradiations, Upton, NY, USA, 7–11 December 1963.

[34] Li X., Lassner M., Zhang Y. (2002). Deleteagene: A fast neutron deletion mutagenesis-based gene knockout system for plants. Int. J. Genom..

[35] Batchelor A., Phillips R.J., Searle A. (1966). A comparison of the mutagenic effectiveness of chronic neutron-and γ-irradiation of mouse spermatogonia. Mutat. Res. Fundam. Mol. Mech. Mutagenesis.

[36] Chakraborty N., Sarkar G.M., Lahiri S.C. (2003). Effect of physical irradiation and chemical mutagen treatment on methane production by methanogenic bacteria. World J. Microbiol. Biotechnol..

[37] Ayaki T., Fujikawa K., Ryo H., Itoh T., Kondo S. (1990). Induced rates of mitotic crossing over and possible mitotic gene conversion per wing anlage cell in Drosophila melanogaster by X rays and fission neutrons. Genetics.

[38] Unrau P. (1986). The relative biological effectiveness of 14.5-MeV neutrons for the induction of gene conversion and mutation in yeast. Radiat. Res..

[39] Smittle B., LaBrecque G., Carroll E. (1971). Comparative effectiveness of fast neutrons and gamma rays in producing sterility in house flies. J. Econ. Entomol..

[40] Tahmisian T.N., Vogel H.H. (1953). Relative Biological Effectiveness of Fast Neutrons, Gamma Rays, X-Rays on Grasshopper Nymph Ovarioles. (*Melanoplus differentialis*). Proc. Soc. Exp. Biol. Med..

[41] Erdman H.E. (1965). Fast-neutron Effects on Productivity of Young and Old Flour Beetles, Tribolium Castaneum. Herbst, and Alterations at Different Temperatures and after Exposure of Either or Both Sexes. Int. J. Radiat. Biol. Relat. Stud. Phys. Chem. Med..

[42] Chen G., Xu Y., Sun Y., Liu J., Wang X. (2011). Effect of fast neutron irradiation on production of cellulase from Trichoderma viride. J. Jilin Agric. Univ..

[43] Gragg R. (1974). Response of Chinese Hamster Ovary Cells to Fast Neutron Radiotherapy Beams. [Gamma Radiation].

[44] Nakamura N., Okada S., Suzuki S., Ito A. (1982). Mutations induced by γ-rays and fast neutrons in cultured mammalian cells. Mutat. Res..

[45] Liu S., Xu J., Chen W., Fu H., Ma L.Y., Xu H., Xinnian L., Wu M., Ma F. (2016). Enhancement of lipid productivity in green microalgae Chlorella sp. via fast neutron irradiation. Biomass Bioenergy.

[46] Mehandjiev A., Kosturkova G., Mihov M. (2001). Enrichment of Pisum sativum gene resources through combined use of physical and chemical mutagens. Israel J. Plant Sci..

[47] Abdel Haliem E., Abdullah H., AL-Huqail A.A. (2013). Oxidative damage and mutagenic potency of fast neutron and UV-B radiation in pollen mother cells and seed yield of *Vicia faba* L. BioMed Res. Int..

[48] Menda N., Semel Y., Peled D., Eshed Y., Zamir D. (2004). In silico screening of a saturated mutation library of tomato. Plant J..

[49] Bolon Y.-T., Stec A.O., Michno J.-M., Roessler J., Bhaskar P.B., Ries L., Dobbels A.A., Campbell B.W., Young N.P., Anderson J.E. (2014). Genome resilience and prevalence of segmental duplications following fast neutron irradiation of soybean. Genetics.

[50] KA S., Larik H. (1982). Persistence of Chromosomal Aberrations in Mutated Populations of *Triticum aestivum*. Cytologia.

[51] Bruggemann E., Handwerger K., Essex C., Storz G. (1996). Analysis of fast neutron-generated mutants at the Arabidopsis thaliana HY4 locus. Plant J..

[52] Bhaskaran S., Swaminathan M. (1960). Polyploidy and radiosensitivity in wheat and barley. Genetica.

[53] Fitzgerald T.L., Powell J.J., Stiller J., Weese T.L., Abe T., Zhao G., Jia J., McIntyre C.L., Li Z., Manners J.M. (2015). An assessment of heavy ion irradiation mutagenesis for reverse genetics in wheat (*Triticum aestivum* L.). PLoS ONE.

[54] Chopra V. (2005). Mutagenesis: Investigating the process and processing the outcome for crop improvement. Curr. Sci. Bangalore.

[55] Krishnaswami R. (1968). The Relationship between Response to Radiations and Nature of Polyploidy in Some Crop Plants. Caryologia.

[56] Chai J.-S., Kim J.-H., Yang T.-G., Lyu J.-I., Lee H.-Y., Yang D.-C., Bae C.-H. (2005). Characteristics of tobacco and rice plants irradiated with neutron beam. Korean J. Plant Resour..

[57] Xiao J., Jia X., Wang H., Zhao R., Fang Y., Gao R., Wu Z., Cao A., Wang J., Xue Z. (2011). A fast-neutron induced chromosome fragment deletion of 3BS in wheat landrace Wangshuibai increased its susceptibility to Fusarium head blight. Chromosome Res..

[58] Hanafy M.S., Mohamed H.A. (2014). Effect of irradiation of wheat grains with fast neutrons on the grain yield and other characteristics of the plants. Appl. Radiat. Isot..

[59] Abe A., Kosugi S., Yoshida K., Natsume S., Takagi H., Kanzaki H., Matsumura H., Yoshida K., Mitsuoka C., Tamiru M. (2012). Genome sequencing reveals agronomically important loci in rice using MutMap. Nat. Biotechnol..

[60] Yugandhar P., Sun Y., Liu L., Negi M., Nallamothu V., Sun S., Neelamraju S., Rai V., Jain A. (2018). Characterization of the loss-of-function mutant NH101 for yield under phosphate deficiency from EMS-induced mutants of rice variety Nagina22. Plan. Physiol. Biochem..

[61] Takagi H., Tamiru M., Abe A., Yoshida K., Uemura A., Yaegashi H., Obara T., Oikawa K., Utsushi H., Kanzaki E. (2015). MutMap accelerates breeding of a salt-tolerant rice cultivar. Nat. Biotechnol..

[62] Sánchez-Martín J., Steuernagel B., Ghosh S., Herren G., Hurni S., Adamski N., Vrána J., Kubaláková M., Krattinger S.G., Wicker T. (2016). Rapid gene isolation in barley and wheat by mutant chromosome sequencing. Genome Biol..

[63] Kaur P., Gaikwad K. (2017). From genomes to GENE-omes: Exome sequencing concept and applications in crop improvement. Front. Plant Sci..

[64] Tadele Z., Chikelu M., Till B.J. (2010). TILLING for mutations in model plants and crops. Molecular Techniques in Crop Improvement.

[65] Sevanthi A.M., Kandwal P., Kale P.B., Prakash C., Ramkumar M., Yadav N., Mahato A.K., Sureshkumar V., Behera M., Deshmukh R.K. (2018). Whole genome characterization of a few EMS-induced mutants of upland rice variety Nagina 22 reveals a staggeringly high frequency of SNPs which show high phenotypic plasticity towards the wild-type. Front. Plant Sci..

[66] Wachsman G., Modliszewski J.L., Valdes M., Benfey P.N. (2017). A simple pipeline for mapping point mutations. Plant Physiol..

[67] Sonah H., Bastien M., Iquira E., Tardivel A., Légaré G., Boyle B., Normandeau É., Laroche J., Larose S., Jean M. (2013). An improved genotyping by sequencing (GBS) approach offering increased versatility and efficiency of SNP discovery and genotyping. PLoS ONE.

[68] Kamolsukyeunyong W., Ruengphayak S., Chumwong P., Kusumawati L., Chaichoompu E., Jamboonsri W., Saensuk C., Phoonsiri K., Toojinda T., Vanavichit A. (2019). Identification of spontaneous mutation for broad-spectrum brown planthopper resistance in a large, long-term fast neutron mutagenized rice population. Rice.

[69] Sikora P., Chawade A., Larsson M., Olsson J., Olsson O. (2011). Mutagenesis as a tool in plant genetics, functional genomics, and breeding. Int. J. Plant Genom..

[70] Anderson J.E., Michno J.-M., Kono T.J., Stec A.O., Campbell B.W., Curtin S.J., Stupar R.M. (2016). Genomic variation and DNA repair associated with soybean transgenesis: A comparison to cultivars and mutagenized plants. BMC Biotechnol..

[71] Novak F., Brunner H. (1992). Plant breeding: Induced mutation technology for crop improvement. IAEA Bull..

[72] Zhong-Hua W., Xin-chen Z., Yu-lin J. (2014). Development and characterization of rice mutants for functional genomics studies and breeding. Rice Sci..

[73] Campbell B.W., Hofstad A.N., Sreekanta S., Fu F., Kono T.J., O’Rourke J.A., Vance C.P., Muehlbauer G.J., Stupar R.M. (2016). Fast neutron-induced structural rearrangements at a soybean NAP1 locus result in gnarled trichomes. Theor. Appl. Genet..

